# The phosphoproteome of choroid plexus epithelial cells following infection with Neisseria meningitidis

**DOI:** 10.3389/fcimb.2023.1113528

**Published:** 2023-03-31

**Authors:** Rosanna Herold, Lea Denzer, Walter Muranyi, Carolin Stump-Guthier, Hiroshi Ishikawa, Horst Schroten, Christian Schwerk

**Affiliations:** ^1^ Pediatric Infectious Diseases, Department of Pediatrics, Medical Faculty Mannheim, Heidelberg University, Mannheim, Germany; ^2^ European Center for Angioscience, Medical Faculty Mannheim, Heidelberg University, Mannheim, Germany; ^3^ Laboratory of Clinical Regenerative Medicine, Department of Neurosurgery, Faculty of Medicine, University of Tsukuba, Tsukuba, Japan

**Keywords:** blood-cerebrospinal fluid barrier, host innate signaling, host pathogen interaction, phosphoproteome, meningitis, *Neisseria meningitidis*

## Abstract

The Gram-negative bacterium *Neisseria meningitidis*, which causes meningitis in humans, has been demonstrated to manipulate or alter host signalling pathways during infection of the central nervous system (CNS). However, these complex signalling networks are not completely understood. We investigate the phosphoproteome of an *in vitro* model of the blood-cerebrospinal fluid barrier (BCSFB) based on human epithelial choroid plexus (CP) papilloma (HIBCPP) cells during infection with the *N. meningitidis* serogroup B strain MC58 in presence and absence of the bacterial capsule. Interestingly, our data demonstrates a stronger impact on the phosphoproteome of the cells by the capsule-deficient mutant of MC58. Using enrichment analyses, potential pathways, molecular processes, biological processes, cellular components and kinases were determined to be regulated as a consequence of *N. meningitidis* infection of the BCSFB. Our data highlight a variety of protein regulations that are altered during infection of CP epithelial cells with N. meningitidis, with the regulation of several pathways and molecular events only being detected after infection with the capsule-deficient mutant. Mass spectrometry proteomics data are available via ProteomeXchange with identifier PXD038560.

## 1 Introduction

Bacterial meningitis, an inflammatory disease of the central nervous system (CNS), is still a disease of global dimension, characterized by the infection of the CNS by invading bacteria. The infection can end fatally or result in long-term neurological sequelae in survivors (Dando et al., 2014).

One of the major barriers protecting the brain from invading pathogens is the blood-cerebrospinal fluid barrier (BCSFB). Pathogens need to interact with and overcome this barrier to enter the CNS and cause meningitis (Dando et al., 2014; Schwerk et al., 2015; Herold et al., 2019). The BCSFB can be separated into barriers to the outer CSF located at the arachnoidea and blood vessels present in the subarachnoidal space, and a barrier to the inner CSF located at the choroid plexus (CP) in the brain ventricles (Weller et al., 2018). The epithelial cells of the CP display polarity, form tight junctions and are the morphological correlates of the inner BCSFB (Dando et al., 2014; Tietz and Engelhardt, 2015).

One of the pathogens demonstrated to infect the CNS *via* the CP are *Neisseria meningitidis* (*N. meningitidis*), human-specific gram-negative bacteria that can colonize the nasopharynx extracellularly, but are often non-pathogenic and commensal (Herold et al., 2019). The polysaccharide capsule of *N. meningitidis* has been described as a major contributor to meningococcal disease. The expression of a capsular polysaccharide enables *N. meningitidis* to survive in the bloodstream, which subsequently enables it to reach the barriers of the CNS. The infection of the host cells by *N. meningitidis* is, however, attenuated by the capsule due to electrostatic repulsion or the covering of other bacterial structures that can facilitate adhesion (St Geme and Falkow, 1991; Hammerschmidt et al., 1996; Kim et al., 2003). Importantly, capsule expression and structure can be switched *in vivo* by phase variation (Tzeng et al., 2016).

After initial attachment to host cells, the hijacking of host post-translational mechanisms, such as protein phosphorylation, has been described as a central strategy for bacterial pathogens during infection, enabling them to subvert host cell function (Ribet and Cossart, 2010). Indeed, *N. meningitidis* was demonstrated to induce host cell signalling events to facilitate invasion into host tissues. Examples of previously described mechanisms include the recruitment of ezrin and the activation of Src kinase and cortactin resulting in a reorganization of the actin cytoskeleton and the formation of membrane protrusions that take up the pathogens (Schubert-Unkmeir, 2017; Borkowski et al., 2020).

Infection with *N. meningitidis* is furthermore accompanied by an acute inflammatory response. The transcription factor NF-κB, associated with the release of proinflammatory cytokines and chemokines during the inflammatory response, was shown to be active in an *in vitro* model of the BCSFB, the human CP papilloma (HIBCPP) cells, after infection with *N. meningitidis* (Borkowski et al., 2014; Herold et al., 2021a). Furthermore, a differential regulation of mitogen-activated protein kinases (MAPK) by wildtype and capsule-deficient *N. meningitidis* serogroup B strains could be demonstrated in HIBCPP cells, wherein *N. meningitidis* wildtype strains required active extracellular signal-regulated kinases (Erk1/2) and p38 pathways for infection, however, the invasion by the capsule-deficient mutant was independent of Erk1/2 and of p38 activity (Herold et al., 2021a). Analysis of the HIBCPP transcriptome after infection with *N. meningitidis* further pointed towards an additional reduction of cellular responses including NFκB and JAK-STAT signalling suggesting a manipulation of different host cell signalling pathways during infection (Herold et al., 2021a).

Phosphoproteomic approaches have enabled a successful profiling of the changes in host protein phosphorylations following the infection by bacterial pathogens such as *Escherichia coli*, *Shigella* and *Salmonella* (Imami et al., 2013; Schmutz et al., 2013; Scholz et al., 2015). To our knowledge, no such analysis has been reported for *N. meningitidis*.

In order to discover signalling pathways that are modulated during infection with *N. meningitidis* or involved in the process of infection, we performed an analysis of the host cell phosphoproteome of HIBCPP cells, as an *in vitro* model of the BCSFB, after infection with *N. meningitidis*. This phosphoproteome analysis gave insight into pathogen-induced changes in intracellular signalling in infected cells which may help uncover new pharmacological targets to prevent *N. meningitidis* infection of the CNS.

## 2 Materials and methods

### 2.1 Bacterial strains and growth conditions

The bacterial strains used in this study were the *N. meningitidis* strain MC58 (WUE2135; (Mcguinness et al., 1991)) and its capsule-deficient mutant MC58siaD (WUE2425; (Ram et al., 2003)). The stock cultures were stored at -80°C in DMEM/F12 supplemented with 1% fetal calf serum (FCS), 5 mg/ml insulin and 15% glycerol. For the infection experiments, the stocks were plated on Chocolate Agar with Vitox (Oxoid, Wesel, Germany) and grown at 37°C in 5% CO_2_ atmosphere overnight. Single colonies of the overnight culture were picked, dissolved and washed in phenol-red free DMEM/F12 with 1% FCS and 5 mg/ml insulin and adjusted to an optical density of 1.0 at 600nm (OD_600_). The suspension, containing approximately 1x10^9^ colony forming units (CFU) per ml, was used to infect the cells and achieve a multiplicity of infection (MOI) of 100. Bacterial growth was monitored throughout all experiments.

### 2.2 Cultivation of HIBCPP on transwell filters and surveillance of barrier function

The HIBCPP (RRID : CVCL_U566) cells were cultured in DMEM/F12 with 10% FCS and 5 mg/ml insulin. For the infection experiments, cells were grown in the inverted cell culture system as previously described (Schwerk et al., 2012). Barrier integrity as well as confluency of the cell layer was determined by measuring the transepithelial electrical resistance (TEER) by using an epithelial voltohmmeter, model Millicell- ERS STX-2 electrode system (Millipore, Schwalbach, Germany). Experiments were performed when a TEER of at least 230 Ω·cm² was reached.

### 2.3 Infection of HIBCPP cells with *N. meningitidis* and generation of protein lysates

Infection of the HIBCPP cells with the *N. meningitidis* strains MC58 and MC58siaD was described previously (Borkowski et al., 2014). In short, the confluent HIBCPP cells were grown in the inverted culture system and infected with the *N. meningitidis* strains at an MOI of 100 for 4h in DMEM/F12 with 1% FCS and 5 mg/ml insulin. Bacterial infection of the HIBCPP cells was followed by a wash step in PBS and subsequent extraction of whole protein lysate by using modified RIPA buffer (1x RIPA lysis buffer, 50 mM NaF, 1 mM Na_3_VO_4_, 1 mM PMSF, protease inhibitor cocktail). These lysates were sent to the DTU Proteomics Core facility at the Technical University of Denmark for analysis of the phosphoproteome. The infection experiment was performed five times and for each condition, protein lysates of three separate infected filters were pooled.

### 2.4 Immunoblot

Whole protein content of an aliquoted amount of the lysates used for the phosphoproteomics analysis was determined using the DC Protein Assay (BioRad, München, Germany) according to the manufacturer´s instructions. 10 µg of protein was resolved by 4–12% Bis–Tris gels (Invitrogen, Karlsruhe, Germany) and electrotransferred onto nitrocellulose membranes. The MAPK p38 and its phosphorylated form p-p38 were detected using the antibodies rabbit anti-phospho-p38 and rabbit anti-p38, which were obtained from Cell Signalling (Cambridge, UK). The Immobilon Western Kit (Millipore, Schwalbach, Germany) was used to visualize immunoreactivity.

### 2.5 Sample preparation for mass-spectrometry

The samples were lysed using a lysis buffer (consisting of 6 M guanidinium hydrochloride, 10 mM tris(2-carboxyethyl) phosphine, 40 mM chloroacetamide, 50 mM HEPES pH 8.5). The samples were then boiled at 95°C for 5 min and subsequently sonicated on the ‘high’ setting for 5 × 30 s in a Bioruptor sonication water bath (Diagenode) at 4°C. The protein concentration was determined using a Pierce Gold BCA kit (Thermo Fisher Scientific) and 500-1000 µg were used for digestion. The samples were diluted 1:3 with 10% acetonitrile, 50 mM HEPES, pH 8.5. In the next step, LysC (mass spectrometry grade, Wako) was added in a 1:50 (enzyme to protein) ratio. Next, the samples were incubated at 37 °C for 4 h. Samples were further diluted to 1:10 using 10% acetonitrile, 50 mM HEPES pH 8.5. Next, trypsin (mass spectrometry grade, Promega) was added in a 1:100 (enzyme to protein) ratio, and samples were incubated overnight at 37 °C. The enzyme activity was quenched by adding 2% trifluoroacetic acid (TFA) to achieve a final concentration of 1%.

### 2.6 StageTip assisted desalting

Prior to the Ti-IMAC enrichment, the peptides were desalted on a SOLAμ C18 plate (Thermo Fisher Scientific). For each of the samples, the C18 material was activated using 200 µl of 100% methanol (HPLC grade, Sigma), and 200 µl of buffer B (80% acetonitrile, 0.1% formic acid). The C18 bedding was subsequently equilibrated twice using 200 µl of buffer A (0.1% formic acid). Afterwards, 500-1000 µg of the sample was loaded using centrifugation at 1,000 r.p.m. The C18 bedding was washed twice with 200 µl of buffer A and the peptides were eluted into clean 1.5 ml Eppendorf tubes using 40% acetonitrile and 0.1% formic acid. The eluted peptides were concentrated in an Eppendorf Speedvac and reconstituted in 50mM HEPES (pH 8.5).

### 2.7 Phospho enrichment

The Ti-IMAC particles were prepared according to modified manufacturers protocol. In short, the particles were agitated. 50 µl of particles per 500 µg of peptide enrichment were then placed in a 2 ml tube, which was then placed on a magnetic rack for 10 s. The shipping solution was removed and particles were washed with 200 µl 70% EtOH followed by 100 µl 1% NH_4_OH and finally 3 times with 100 µl 80% Acetonitrile 1M glycoltic acid, 5% TFA, each time gently agitating for 1-5 min. Afterwards, the samples were diluted 1:1 with 80% Acetonitrile, 1M glycolic acid, 5% TFA and added to the equilibrated particles. The suspension was incubated at RT for 30 min with agitation. The bound peptides were washed 2 times with 400 µl 80% Acetonitrile, 1% TFA, followed by two wash steps in 400 µl 10% Acetonitrile, 0.1% TFA. The enriched phosphopeptides were eluted 3 times with 80 µl of NH_4_OH, each time agitating for 20 min at RT. The peptides were collected in a new 0.5 ml Protein-LoBind tube and Speed-vaced for 30min at 60°C to remove most of the NH_4_OH. Samples were acidified using 10% TFA and subjected to a C18 desalting step. Dried peptides were resuspended in 100 mM tetraethylammonium bromide (pH 8.5) for TMT labelling.

### 2.8 TMT labelling

Labelling was done according to the manufacturer’s instructions, and labelled peptides were subsequently mixed 1:1:1:1:1:1:1:1:1:1:1:1:1:1:1:1 (16-plex), acidified to 1% TFA. The acetonitrile concentration was brought down to <5% with the use of 2% TFA. Before the mass spectrometry analysis, the peptides were fractionated using an offline Thermo Fischer Ultimate 3000 liquid chromatography system applying the high pH fractionation (5 mM ammonium bicarbonate, pH 10) at a flow rate of 5 µL min^-1^. Afterwards, 20 µg of peptides were separated over a 120 min gradient (from 5% to 35% acetonitrile), with fractions being collected every 120s. The resulting 60 fractions were pooled into 30 final fractions (fractions 1 + 31, 2 + 32, 3 + 33, and so on), acidified to pH <2 with 1% TFA and loaded onto EvoSep StageTips according to the manufacturer’s protocol.

### 2.9 Global TMT-labelled liquid chromatography–mass spectrometry data acquisition

For each of the fractions, peptides were analyzed using the preset ‘30 samples per day’ method on the EvoSep One instrument. Peptides were eluted over a 44 min gradient and analyzed on an Exploris 480 instrument (Thermo Fisher Scientific) running in a data-dependent tandem mass spectrometry top-speed mode (3 s cycle time). Full mass spectrometry spectra were collected at a resolution of 60,000, with a normalized automatic gain control target of 300% and automatic maximum injection time, using a scan range of 375–1,500 m/z. The tandem mass spectrometry spectra were obtained at a resolution of 30,000 with the ‘turboTMT’ functionality enabled, with a normalized automatic gain control target of 100% and automatic maximum injection time, a normalized collision energy of 33 and an intensity threshold of 8e^3^. The first mass was set to 120 m/z to ensure capture of the TMT reporter ions. Precursors were isolated with a 0.7 m/z isolation window and Precursor Fit enabled to 70% at a window of 0.7 m/z. Dynamic exclusion was set to 60 s, and ions with a charge state<2, >7 and unknown were excluded. Mass spectrometry performance was verified for consistency by running complex cell lysate quality-control standards. The chromatography was monitored to check for reproducibility.

### 2.10 TMT quantitative proteomics analysis

Raw files were analyzed using Proteome Discoverer 2.4. TMTPro reporter ion quantitation was enabled in the processing and consensus steps, and spectra were matched against the *Homo sapiens* reference proteome (UP000005640, last modified October 18, 2020, protein count 75776) obtained from Uniprot. Dynamic modifications were set as phosphorylation (S,T,Y), oxidation (M), deamidation (N,Q) and acetyl on protein N termini. Cysteine carbamidomethyl (on C residues) and TMTPro (on peptide N termini and K residues) were set as static modifications. All results were filtered to a 1% false discovery rate. TMT reporter ion quantitation done using the built-in ‘reporter ions quantifier’ node.

### 2.1 Statistical analysis

Prior to statistical analysis, the data was filtered so that only the phosphorylated peptides that were found in at least 75% of the samples or found in 60% in at least one sample group were included in the analysis. In this analysis the filtering resulted in a total of 3767 quantifiable peptides being used for the statistical testing. Each expression was tested for normal distribution by Shapiro-Wilk test. If a p-value > 0.05 was obtained (i.e. the expression is exhibiting a normal distribution), the groups were compared using a parametric test such as limma (Ritchie et al., 2015) which is a moderated Analysis of Variance (ANOVA) test, followed by a Benjamini Hochberg p-value correction. If the obtained p-value was below 0.05, a non-parametric statistical test was performed (Wilcoxon-Mann-Whitney test, referred to in the text as Wilcoxon test). The data is summarized in the **Supplementary Tables 1–3**. A total of 11301 comparisons were tested, 2207 of which had a p-value below or equal to 0.05. When adjusting the p-value for multiple comparisons using the Benjamini-Hochberg false discovery rate procedure, 534 passed the filtration. Of these, 534 had a regulation of more than 30% and 186 have an absolute log_2_ fold change higher or equal to 1. The regulations are distributed among 3 group comparisons.

Enrichment analyses were performed using the Reactome database (RRID : SCR_019316) (Yu and He, 2016). This analysis allows for the identification of cellular components, molecular function, biological processes kinases and pathways that are enriched more than anticipated by chance from a gene list based on the phosphorylated peptides. Enrichment Analyses were carried out using all regulations with an uncorrected p-value below 0.05 in the group wise statistical analysis. This analysis was carried out using the Benjamini-Hochberg Procedure and returning the enriched pathways. The adjusted p-value cut off was set at 0.05. Tables with the identified enriched pathways were constructed for all group comparisons and can be found in **Supplementary Table 4**.

These enrichment analyses provide information on aspects such the cellular location where the phosphorylated peptides execute their functions, molecular activities where they are involved, and the biological processes where they might be involved. These analyses were performed using clusterProfiler (RRID : SCR_016884) (Yu et al., 2012) and DOSE (R/Bioconductor package, RRID : SCR_006442) (Yu et al., 2015). For the kinase activity enrichment analysis, the KSEA App (Wiredja et al., 2017) and Signor2 knowledge base (Licata et al., 2020) were used.

## 3 Results

In order to investigate the influence of the *N. meningitidis* strains on the signal transduction in HIBCPP cells, a phosphoproteomics analysis of the cells after infection with the *N. meningitidis* serogroup B wildtype strain (MC58) and the capsule-deficient mutant (MC58siaD) was carried out in comparison to uninfected HIBCPP cells. For this purpose, the HIBCPP cells were infected in the inverted culture for 4 hours at an MOI of 100 and lysed with the aid of the modified RIPA buffer. We have previously shown that after 4 hours infection from the basolateral side *N. meningitidis* has invaded into HIBCPP cells (Schwerk et al., 2012; Borkowski et al., 2014), and phosphorylation of Erk1/2 and p38 was observed using an MOI of 100 (Herold et al., 2021a). Noteworthy, only a small proportion (around 0.1%) of the meningococcal population is internalized. The samples were processed and analyzed by the DTU Proteomics Core facility at the Technical University of Denmark for the phosphoproteome analysis, and data analysis was performed by Biogenity (Denmark). Sample preparation, mass spectrometric analysis and statistical analysis are described in detail in the Material and Methods section.

### 3.1 Infection of HIBCPP cells with *N. meningitidis* alters the host cells phosphoproteome

**Figure 1:**
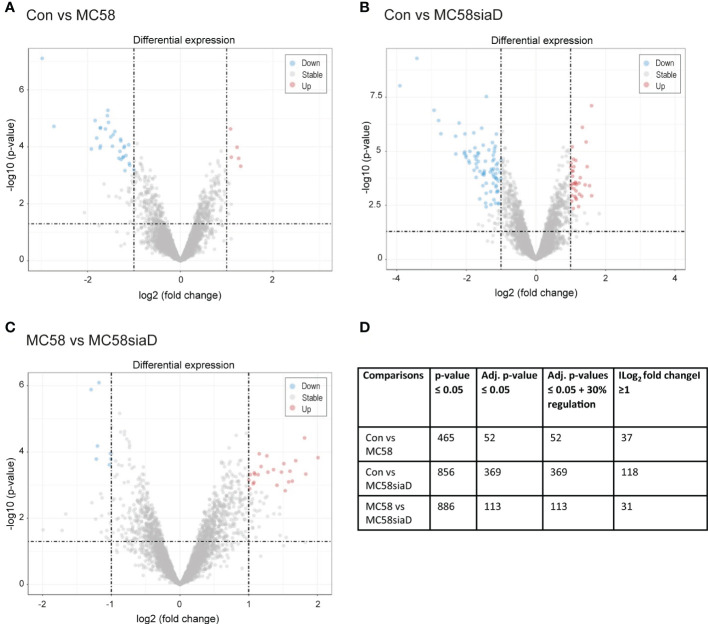
Volcano plots visualizing phosphorylated peptides with larger fold changes and statistical significance. The p-values were adjusted using the Benjamini-Hochberg procedure for limma and Wilcoxon test. The plots depict the comparisons Con vs MC58 **(A)** and Con vs MC58siaD **(B)** as well as MC58 vs MC58siaD **(C)**. The dashed horizontal lines show where the unadjusted p-value is equal to 0.05, with points above the line corresponding to p-values > 0.05. The vertical dashed lines show where the log_2_ fold change is equal to -1 and 1. All statistically significant regulations (adjusted p-value < 0.05) with log_2_ fold changes greater than one are shown as colored dots (red and blue). Grey dots represent the remaining fold changes. The blue dots represent statistically significant log_2_ fold changes < -1, red dots represent statistically significant log_2_ fold changes > 1. **(D)** summarizes the number of regulations with a p-value ≤0.05, regulations remaining after p-values were adjusted for multiple comparisons using the Benjamini-Hochberg procedure, as well as the regulations with a log_2_ fold change ≥1 and adjusted p-values ≤ 0.05 + 30% regulation.

**Figure 1** depicts the volcano plots of the phosphorylations that were significantly regulated according to the bioinformatic analysis of the data. The volcano plots were constructed to facilitate the visualization of the highly significant phosphorylations with larger fold changes and statistical significance of the comparisons “Con vs MC58” (**Figure 1A**) and “Con vs MC58siaD” (**Figure 1B**) as well as “MC58 vs MC58siaD” (**Figure 1C**). P-values were adjusted using the Benjamini-Hochberg procedure for limma and Wilcoxon test. The dashed horizontal line shows where the unadjusted p-value is equal to 0.05, with points below the line corresponding to p-values > 0.05 and the points above the line correspond to p-values < 0.05. The vertical dashed lines show where the log_2_ fold change is equal to -1 and 1, respectively.

All statistically significant phosphorylated peptides with adjusted p-values < 0.05 and a log_2_ fold changes greater than one are shown as colored dots (red and blue), representing statistically significant log_2_ fold changes < -1 (blue), corresponding to phosphorylated peptides with negative regulations, or statistically significant log_2_ fold changes > 1 (red), corresponding to phosphorylated peptides with positive regulations. **Figures 1A, B** show that the infection with the capsule-deficient mutant of *N. meningitidis* resulted in a higher amount of significant regulations than infection with the wildtype strain. In addition, **Figure 1C** confirms a high overlap of statistically relevant regulations between HIBCPP cells infected with both *N. meningitidis* strains as well as some differentially regulated peptides between both strains.

**Figure 1D** summarizes the results of the statistical analysis of the data. Tables summarizing the regulations before statistical analysis, regulations with p-values ≤0.5 and regulations with adjusted p-values ≤0.05 are shown in **Supplementary Tables 1**–**3**.

### 3.2 Comparison of the effects of *N. meningitidis* wildtype and capsule-deficient mutant on the number of regulated proteins

**Figure 2:**
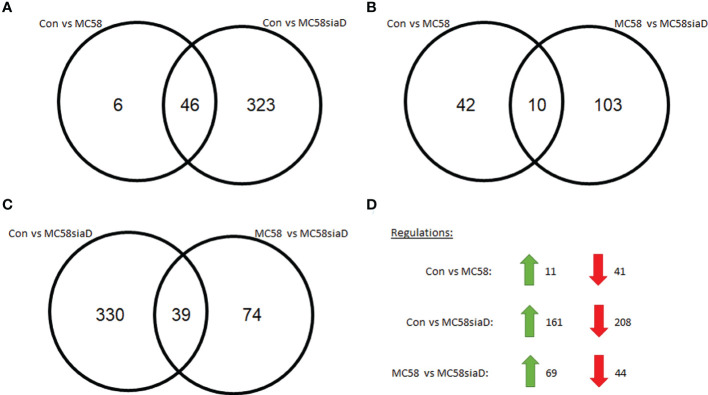
Schematic representation of the number of statistically relevant regulations identified in the phosphoproteomics analysis of HIBCPP infected with *N. meningitidis* wildtype strain and capsule-deficient mutant. Regulations were determined by comparing uninfected HIBCPP cells with cells infected with the *N. meningitidis* wildtype strain (Con vs MC58), uninfected HIBCPP cells with cells infected with the capsule-deficient mutant (Con vs MC58siaD), or cells infected with the *N. meningitidis* wildtype strain with cells infected with the capsule-deficient mutant (MC58 vs MC58siaD). Venn diagrams show overlaps between Con vs MC58 and Con vs MC58siaD **(A)**, between Con vs MC58 and MC58 vs MC58 siaD **(B)**, and Con vs MC58siaD and MC58 vs MC58siaD **(C)**. The distribution of regulations found for Con vs MC58, for Con vs MC58siaD, and for MC58 vs MC58siaD into up-regulated and down-regulated is shown in **(D)**. Only regulations having an adjusted p-value greater than or equal to 0.05 were used.

**Figures 2A–C** show Venn diagrams comparing the impact of infection of HIBCPP cells with the wildtype strain and the capsule-deficient mutant using the regulations with an adjusted p-value <0.05. **Figure 2A** indicates a stronger impact of the capsule-deficient mutant on the host cell phosphoproteome than the wildtype strain. A total of 323 regulations are only identified after infection with the capsule-deficient mutant compared to 6 regulations only found after infection with the wildtype strain (**Figure 2A**). Interestingly, most regulations identified after infection with the wildtype strain can be identified after infection with the capsule-deficient mutant as well, with an overlap of 46 regulations between both strains (**Figure 2A**). Furthermore, little overlap in regulations is found when comparing control cells with infected cells (Con vs MC58 and Con vs MC58siaD) to the cells infected with the wildtype strain and capsule-deficient mutant (MC58 vs MC58siaD) (**Figures 2B, C**).

Furthermore, in a comparison of the untreated controls with the HIBCPP cells infected with MC58, 52 regulations were detected that, according to the statistical analysis, had an adjusted p-value greater than or equal to 0.05. 11 of these regulations showed a positive log_2_ fold-change, while 41 regulations showed a negative fold-change and were therefore downregulated (**Figure 2D**). The comparison of the untreated controls with the HIBCPP cells infected with the capsule-deficient mutant revealed, according to statistical analysis, 369 regulations with an adjusted p-value less than or equal to 0.05. Of these, 161 were upregulated in control cells compared to infected cells while 208 were downregulated (**Figure 2D**). When both conditions were compared in which the HIBCPP cells were infected with the wildtype strain of *N. meningitidis* or its capsule-deficient mutant, 113 regulations were found with an adjusted p-value greater than or equal to 0.05, of which 69 were more strongly upregulated and another 44 were downregulated in cells infected with MC58 compared to cells infected with MC58siaD (**Figure 2D**).

### 3.3 Enrichment analyses of regulated phosphorylations after infections of HIBCPP cells with *N. meningitidis*

In order to translate this primary level of information of the regulated phosphorylations into biological knowledge, enrichment analyses were carried out using all regulations with an unadjusted p-value below 0.05. The enrichment analyses were carried out as described in the Materials and Methods section. Tables summarizing the output of the analyses can be found in the **Supplementary Table 4**.

Dot plots were constructed to illustrate the comparison of the annotated cellular components, molecular functions, biological processes, pathways and kinase enrichment in the different sample group tests. For each enrichment analysis, the three comparisons (Con vs MC58, Con vs MC58siaD, MC58 vs MC58siaD) showed enriched terms. The figures were made using the tools and procedures described in (Yu et al., 2012; Yu et al., 2015; Yu and He, 2016).

#### 3.3.1 Cellular components

**Figure 3:**
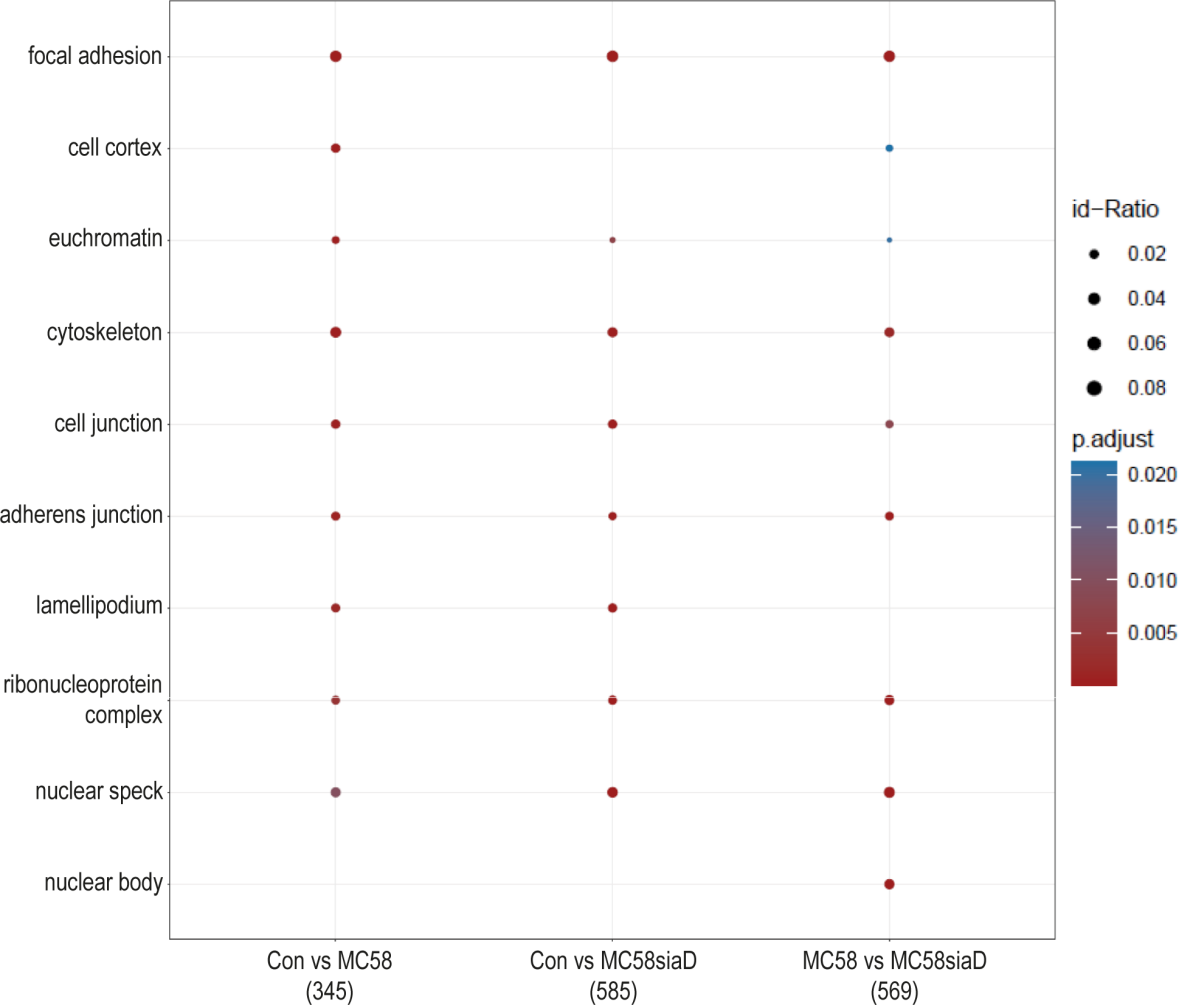
Enrichment analysis of cellular components after infection with *N. meningitidis* wildtype and its capsule-deficient mutant. Enrichment analysis of cellular components was performed using regulations with an uncorrected p-value less than 0.05. Listed are the results of the comparisons of control cells with the wildtype strain (Con vs MC58), the control cells with the capsule-deficient mutant (Con vs MC58siaD), and cells infected with the two strains with each other (MC58 vs MC58siaD). The x-axis depicts the different sample group tests (with the number of genes used for each enrichment analysis within brackets). The y-axis shows the cellular components which were annotated, ranked by the lowest adjusted p-value. Moreover, the color of the dots is scaled by the adjusted p-value from the enrichment analysis, the size of the dots is scaled by the ratio of regulated expressions annotated to each term.

In the enrichment analysis of “cellular components” (**Figure 3**), the effect of infection with the two *N. meningitidis* strains was compared to the uninfected controls, as well as the effect of infection with the wildtype strain on phosphorylations in HIBCPP cells compared to the effect of infection with the capsule-deficient mutant. A large number of the cellular component termini listed in **Figure 3** were identified after infection with both the wildtype and the capsule-deficient mutant. Among the terms found after infection with both strains are components of the cytoskeleton, the cell-cell contacts and the nuclear organization.

#### 3.3.2 Molecular functions

**Figure 4:**
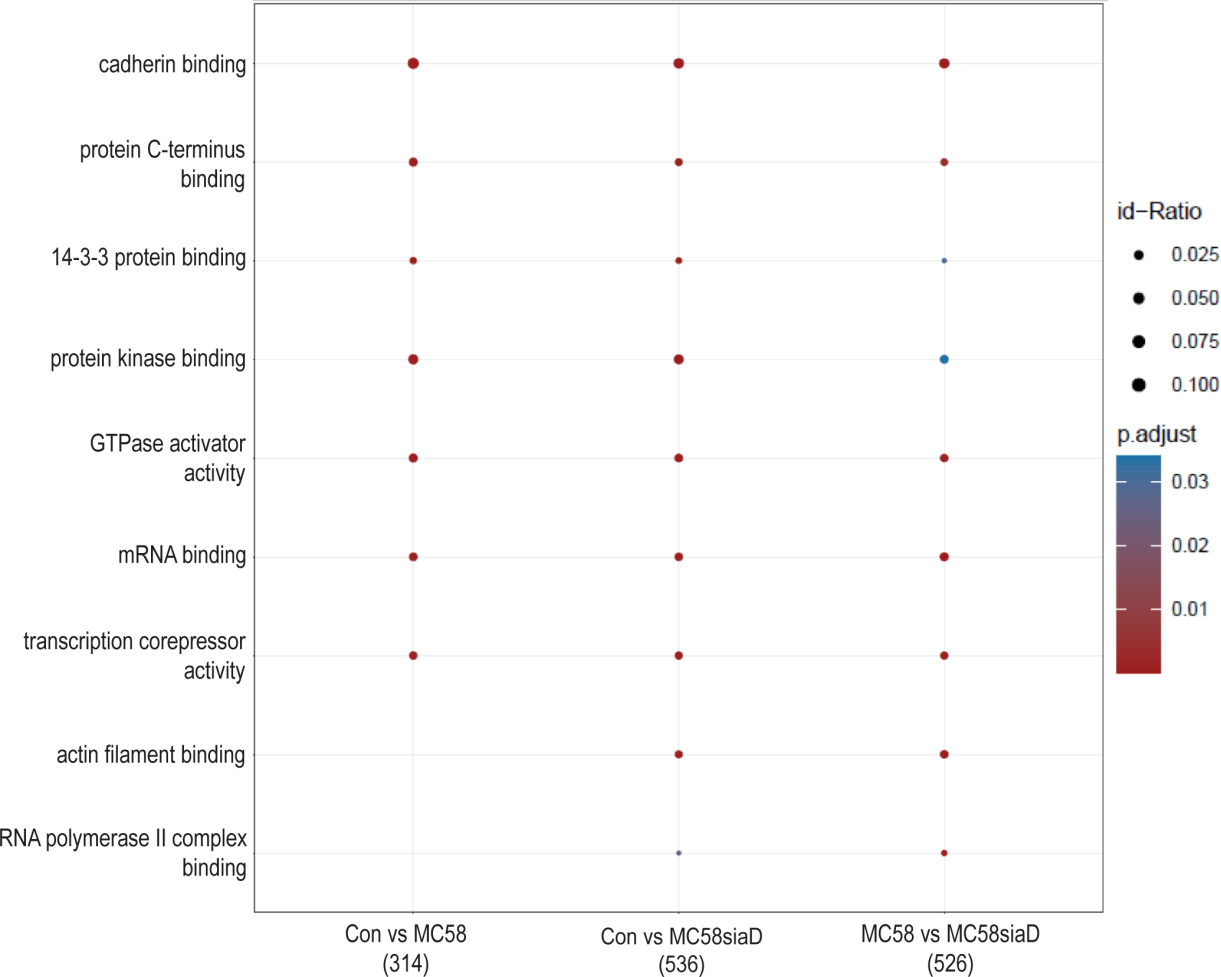
Enrichment analysis of molecular functions after infection with *N. meningitidis* wildtype and its capsule-deficient mutant. Enrichment analysis of molecular functions was performed using regulations with an uncorrected p-value less than 0.05. Listed are the results of the comparisons of control cells with the wildtype strain (Con vs MC58), the control cells with the capsule-deficient mutant (Con vs MC58siaD), and cells infected with the two strains with each other (MC58 vs MC58siaD). The x-axis depicts the different sample group tests (with the number of genes used for each enrichment analysis within brackets). The y-axis shows the molecular functions which were annotated, ranked by the lowest adjusted p-value. Moreover, the color of the dots is scaled by the adjusted p-value from the enrichment analysis, the size of the dots is scaled by the ratio of regulated expressions annotated to each term.

Furthermore, an enrichment analysis of “molecular function” after infection of HIBCPP cells with the *N. meningitidis* strains was performed (**Figure 4**). Among the terms with the highest statistical significance found in all three groups are “cadherin binding”, “protein C-terminus binding”, “protein kinase binding” as well as “14-3-3 protein binding” and several terms pointing towards an impact on the regulation of transcription. Terms only found after infection with the capsule-deficient mutant describe “RNA polymerase II complex binding” and “actin filament binding”.

#### 3.3.3 Biological processes

**Figure 5:**
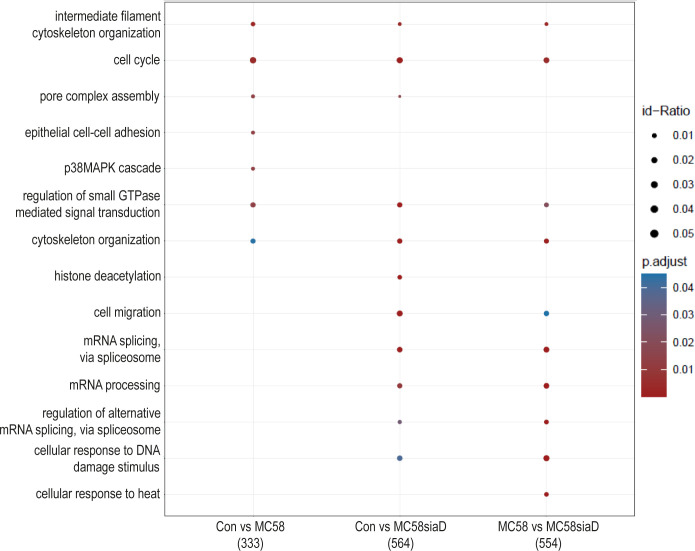
Enrichment analysis of biological processes after infection with *N. meningitidis* wildtype and its capsule-deficient mutant. Enrichment analysis of biological processes was performed using regulations with an uncorrected p-value less than 0.05. Listed are the results of the comparisons of control cells with the wildtype strain (Con vs MC58), the control cells with the capsule-deficient mutant (Con vs MC58siaD), and cells infected with the two strains with each other (MC58 vs MC58siaD). The x-axis depicts the different sample group tests (with the number of genes used for each enrichment analysis within brackets). The y-axis shows the biological processes which were annotated, ranked by the lowest adjusted p-value. Moreover, the color of the dots is scaled by the adjusted p-value from the enrichment analysis, the size of the dots is scaled by the ratio of regulated expressions annotated to each term.

The effect of infection of the *N. meningitidis* strains on the “biological processes” of the HIBCPP cells was also determined using an enrichment analysis of the phosphoproteomics data (**Figure 5**). The comparison of control cells to the cells infected with the *N. meningitidis* wildtype strain provided terms describing an overall regulation of the cytoskeleton and regulation of intermediate filament cytoskeleton organization as well as epithelial cell-cell adhesion. Furthermore, regulation of the “pore complex assembly”, and “cell cycle” as well as a regulation of signaling pathways were found. Control cells compared to cells infected with the capsule-deficient mutant overlapped in most of these terms found for the comparison “Con vs MC58” and revealed further terms pointing towards altered regulation of “mRNA processing”, “histone deacetylation”, “cell migration” and cellular responses to DNA damage and heat after infection.

#### 3.3.4 Signaling pathways

**Figure 6:**
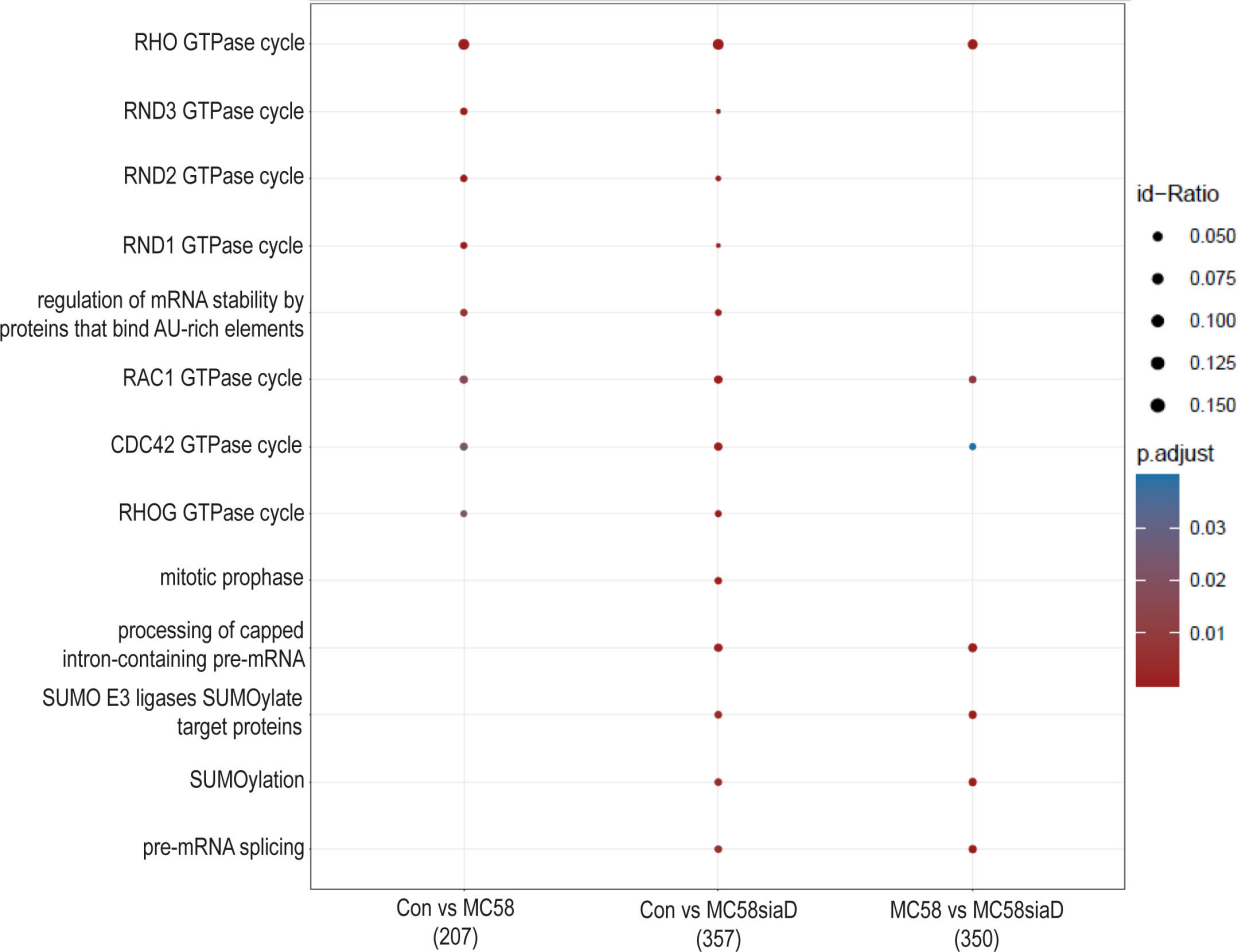
Pathway enrichment analysis after infection with *N. meningitidis* wildtype and its capsule-deficient mutant. Enrichment analysis of pathways was performed using regulations with an uncorrected p-value less than 0.05. Listed are the results of the comparisons of control cells with the wildtype strain (Con vs MC58), the control cells with the capsule-deficient mutant (Con vs MC58siaD), and cells infected with the two strains with each other (MC58 vs MC58siaD). The x-axis depicts the different sample group tests (with the number of genes used for each enrichment analysis within brackets). The y-axis shows the pathways which were annotated, ranked by the lowest adjusted p-value. Moreover, the color of the dots is scaled by the adjusted p-value from the enrichment analysis, the size of the dots is scaled by the ratio of regulated expressions annotated to each term.

An enrichment analysis of “pathways” was performed after infection of HIBCPP cells with the *N. meningitidis* strains compared to untreated controls. A comparison of the annotated signaling pathways that were determined in the various sample groups is summarized in **Figure 6**. The annotated signaling pathways after infection of HIBCPP cells with the *N. meningitidis* wildtype strain and the capsule-deficient mutant point towards altered regulation of GTPases during infection as well as the “regulation of mRNA stability by proteins that bind AU-rich elements”. Analysis of the regulations found after infection with the capsule-deficient mutant also resulted in termini that were not determined in the analysis of the regulated phosphorylations by the wildtype strain. These include terms describing processing of mRNA, a regulation of SUMO E3 ligases and the term “mitotic prophase”.

#### 3.3.5 Kinase enrichment

**Figure 7:**
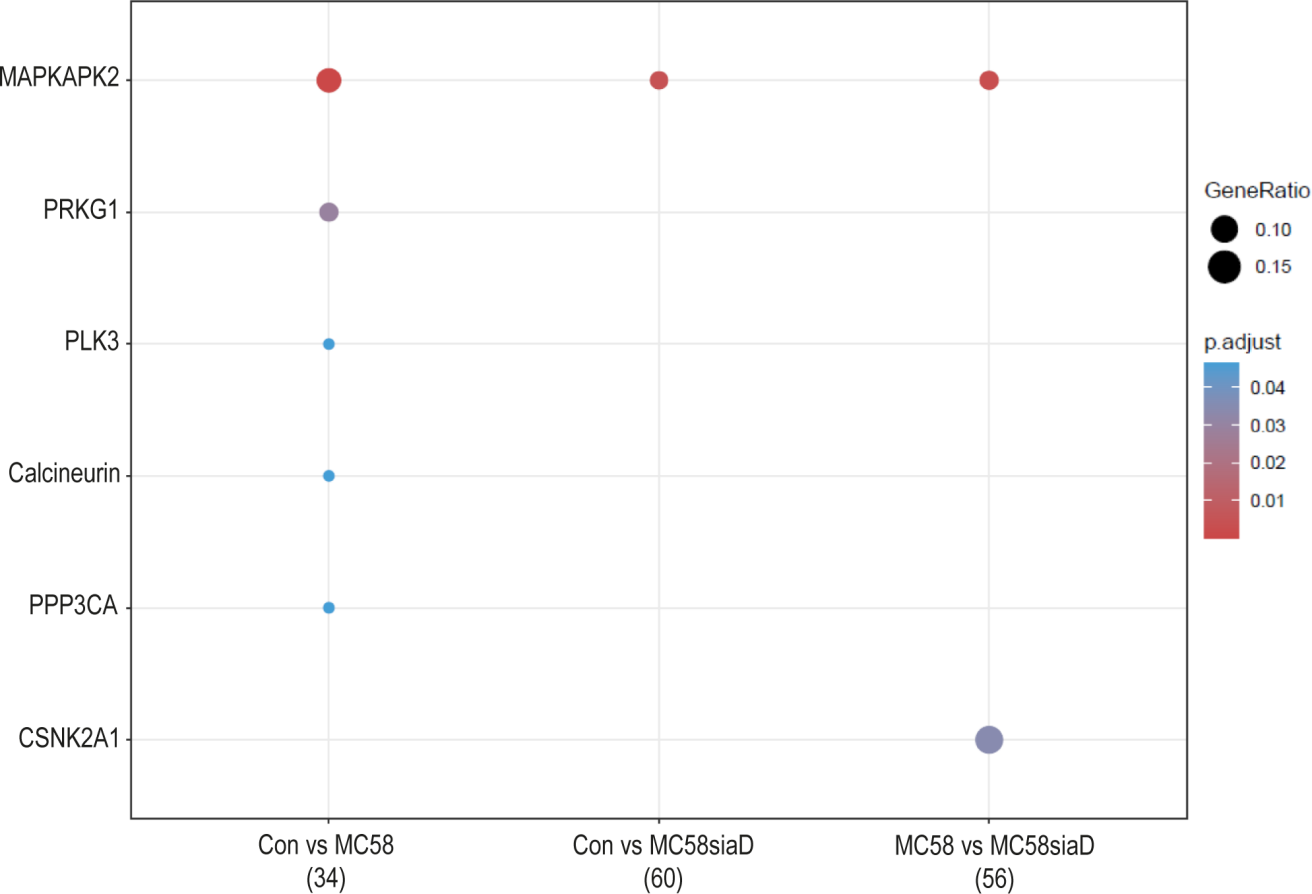
Kinase annotation of enrichment analysis after infection with *N. meningitidis* wildtype and its capsule-deficient mutant. Enrichment analysis of kinases was performed using regulations with an uncorrected p-value less than 0.05. Listed are the results of the comparisons of control cells with the wildtype strain (Con vs MC58), the control cells with the capsule-deficient mutant (Con vs MC58siaD), and cells infected with the two strains with each other (MC58 vs MC58siaD). The x-axis depicts the different sample group tests (with the number of genes used for each enrichment analysis within brackets). The y-axis shows the kinases which were annotated, ranked by the lowest adjusted p-value. Moreover, the color of the dots is scaled by the adjusted p-value from the enrichment analysis, the size of the dots is scaled by the ratio of regulated expressions annotated to each term.

Lastly, an enrichment analysis of the regulated kinases was carried out for all comparisons. **Figure 7** shows the comparison of infection of HIBCPP cells with the two *N. meningitidis* strains in terms of enrichment of various kinases, as well as the comparison of infection with wildtype and capsule-deficient mutant to each other. Almost all of the listed kinases were regulated by the *N. meningitidis* wildtype strain, with the exception of MAPKAPK2, which was found in all three comparisons as well as CSNK2A1, which is only found in the comparison “MC58 vs MC58siaD”.

### 3.4 Kinase activity analysis after infection of HIBCPP cells with *N. meningitidis*

**Figure 8:**
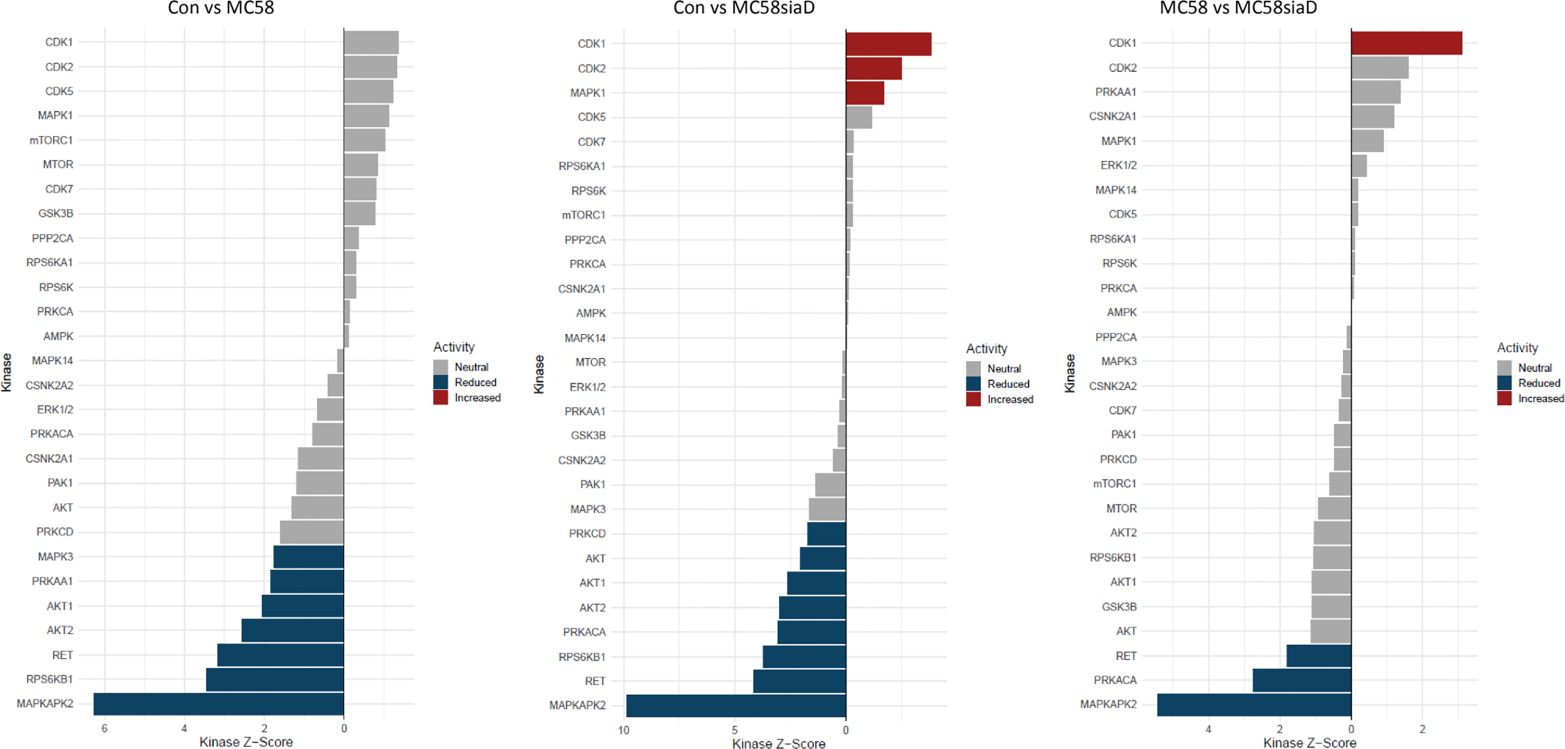
Waterfall plot depicting the kinase activity analysis after infection with *N. meningitidis* wildtype and its capsule-deficient mutant. The kinases on the y-axis are ranked by the changes in the phosphorylation sites that are known to be downstream of that specific kinase, the x-axis depicts the kinases respective activity score. The bars are colored if they have a p-value below 0.05, the blue bars depict reduced activity and the red bars signify increased activity. The figure has been filtered to only include kinases with at least 5 phosphorylated sites mapped to it from the phosphoproteomic data.

The waterfall plots shown in **Figure 8** depict the kinase activity enrichment analysis which was created as described in (Wiredja et al., 2017). The kinases are ranked from top to bottom by the changes in the phosphorylation sites that are known to be downstream of that specific kinase from the Signor2 (Licata et al., 2020) knowledge base. **Figure 8** has been filtered to only include kinases with at least 5 phosphorylated sites mapped to it from the data.

Overall, the kinase activity depicts more potentially regulated kinases after infection of HIBCPP cells with the *N. meningitidis* capsule-deficient mutant than after infection the with *N. meningitidis* wildtype strain. Of note are the kinases CDK1, CDK2 and MAPK1 which are upregulated in control cells in comparison to cells infected with the capsule-deficient mutant. Moreover, kinases found to be upregulated by the infection of HIBCPP cells with both *N. meningitidis* strains in this analysis (downregulated in control cells) include MAPKAPK2, RPS6KB1, RET as well as AKT1 and AKT2, whereas PRKACA activity was upregulated only by MC58siaD.

The enrichment analysis of biological processes includes the term p38 MAPK cascade and the kinase activity analysis includes a positive regulation of MAPKAPK2, which is a downstream target of the kinase p38. We therefore tested the samples that were sent to be analyzed in this study for phosphorylation of p38 for all biological replicates (**Supplementary Figure 5**). The phosphorylation of both the MAPK p38 and Erk1/2 has previously been demonstrated in HIBCPP cells after infection with *N. meningitidis* (Herold et al., 2021a) and is shown here to be stronger after infection with the capsule-deficient mutant in all biological replicates.

## 4 Discussion

The CP represents a barrier between the bloodstream and the CSF. Thus, in the event of a systemic infection, bacteria come into contact with the CP epithelial cells *via* the bloodstream and have to overcome them. With the help of an inverted cell culture model of the HIBCPP cells (Schwerk et al., 2012; Dinner et al., 2016) the contact of the pathogen with the basolateral (bloodstream facing) side of the CP epithelial cells can be examined *in vitro* in order to further characterize the role of BCSFB during bacterial meningitis.

Protein phosphorylation, which acts as a reversible molecular switch, provides a mechanism for controlling protein function during almost all cellular processes and is essential for the rapid response of cells to internal and external cues. To our knowledge, no phosphoproteome analysis has been performed to analyze the effects of *Neisseria* infection at the CP. Consequently, the phosphoproteome of the HIBCPP cells was examined in the untreated state and after four hours of infection with the *N. meningitidis* wildtype strain and its capsule-deficient mutant. The acquired data, although further experimental confirmation is required in the future, serves as a starting point for analysis of molecular events taking place at the CP during infection with *N. meningitidis*.

Statistical analysis of the datasets gives insights into both the phosphorylations identified as a consequence of the infection with *N. meningitidis* as well as potential cellular processes involved in the infection and cellular response identified by enrichment analyses. Importantly this data will need to be verified by functional analysis of the individual targets in future analyses to confirm the involvement of the proteins during infection.

When comparing the statistically significant regulations after infection of HIBCPP cells with the *N. meningitidis* wildtype strain and its capsule-deficient mutant, a stronger impact of the capsule-deficient mutant on the number of regulations can be observed. Furthermore, these regulations overlap in large parts with those identified after infection with the wildtype strain, indicating similar mechanisms of infection between the two strains, but an overall stronger impact on the host cells phosphoproteome after infection with the capsule-deficient mutant. Still, several phosphorylations were only observed after infection with the mutant strain. This observation correlates with the higher rates of infection in HIBCPP cells observed for the mutant strain in the past (Borkowski et al., 2014; Herold et al., 2021a; Herold et al., 2021b). In the enrichment analyses of the datasets, most of the identified terms for each analysis overlap for both strains, indicating that the additional phosphorylations caused by the mutant strain contribute to the same terms.

The enrichment analysis of “cellular components” highlights the impact of infection of HIBCPP cells with both *N. meningitidis* strains on “focal adhesion”, “cell junction” and the “cytoskeleton” of the host cells. In agreement, an interplay between the focal adhesion kinase, cortactin and Src kinase facilitates infection of *N. meningitidis* into human brain microvascular endothelial cells by inducing a cytoskeletal rearrangement and uptake of the pathogen (Slanina et al., 2012). The intestinal pathogens enterohemorrhagic *Escherichia coli* (EHEC) and enteropathogenic *Escherichia coli* (EPEC) also alter a variety of host cell components, including the host cell cytoskeleton and tight junctions (Guttman et al., 2007). Still, in contrast to *N. meningitidis*, EHEC and EPEC possess type III secretion systems for delivery of bacterial effector proteins into host cells (Gaytan et al., 2016), indicating that possibly different mechanisms are responsible. Finally, detection of the terms “ribonucleoprotein complex” and “nuclear speck” indicate an impact on RNA metabolism.

The enrichment analysis of “molecular processes” points towards terms regulated by both the wildtype strain and capsule-deficient mutant describing an impact of the infection on the regulation of “cadherin binding”. Overcoming protective barriers of the host by affecting adherens junctions components of the epithelial cells of these barriers, which include cadherin, has already been described (Devaux et al., 2019). Along these lines, the term “actin filament binding” is also found following infection with the mutant. Other terms as “mRNA binding”, “transcription corepressor activity” and “RNA polymerase II complex binding” indicate a manipulation of gene expression. Noteworthy, it has already been described that secretion of various effectors enables bacteria to not only penetrate the host cell, but also to manipulate their gene expression in order to bypass the host’s immune response (Denzer et al., 2020). Furthermore, pathogens can influence gene expression at the level of mRNA stability, either by disrupting mRNA stabilization or mRNA degradation, as well as translation or protein stability, including the mechanisms of protein activation and protein degradation (Denzer et al., 2020).

The enrichment analysis of “biological processes” strongly highlights that terms describing the manipulation of gene expression, including mRNA splicing, during infection are found, especially following infection with the capsule-deficient mutant of *N. meningitidis*, but not the wildtype strain. The difference in appearance of these biological processes following infection with wildtype or mutant, respectively, opens up the possibility of different mechanisms of infection by both *N. meningitidis* strains. Furthermore, regulated biological processes detected after infection with both strains indicate a manipulation of “cytoskeletal organization” and “intermediate filament cytoskeleton organization”, whereas “epithelial cell-cell adhesion” was only affected by the wildtype. Deregulation of the cytoskeleton has already been described during infection of the BBB by *N. meningitidis* as well as a re-localization of proteins to the cell surface during infection (Slanina et al., 2010; Slanina et al., 2012; Doran et al., 2016; Maissa et al., 2017), and could give further insight into infection mechanisms at the BCSFB, where a differential regulation of proteins known to interact with the actin cytoskeleton has also been described after infection with *N. meningitidis* (Herold et al., 2021b). Of note, the enrichment analysis also pointed towards regulation of the p38 MAPK cascade after infection with the wildtype strain. A manipulation of MAPK signaling pathways by pathogens has previously been demonstrated for the intracellular pathogen *Listeria monocytogenes* and *N. meningitidis* in HIBCPP cells. During these infection experiments both *L. monocytogenes* and *N. meningitidis* activate Erk1/2 and p38 phosphorylation (Dinner et al., 2017; Herold et al., 2021a). However, it needs to be clarified whether similar or different mechanisms are responsible for activation of MAPK signaling.

Enrichment analyses of the phosphoproteomics data were also used to identify potential signaling “pathways” regulated during infection. As described for “biological processes”, terms pointing to regulation of mRNA processing were preferentially found following infection with the mutant. Interestingly, infection with the wildtype strain as well as the capsule-deficient mutant led to identification of a term describing the regulation of mRNA stability by proteins that bind adenine and uridine-rich elements (ARE). These AREs are assigned an important function in the control of the mRNA stability. Changes in mRNA transcription and stability due to these regulatory mechanisms are of great importance for the immune response of the cells as well as the stress response. A deregulation of these mechanisms and the resulting change in the host cell’s transcriptome could be used by pathogens to their advantage (Gingerich et al., 2004).

Collectively, the enrichment analyses point to an impact of both the wildtype and the capsule-depleted mutant on cellular junctions and organization of the host cell cytoskeleton, processes which are involved during invasion of the bacteria into the host cells. Mechanisms of RNA metabolism, including mRNA splicing, are mainly found regulated by the mutant, which might be related to the higher invasion rate of Mc58siaD. Also, evidence for the regulation of MAPK signaling, especially by p38, was found. The enrichment analysis of the accumulation of the kinases as well as the kinase activity enrichment analysis further highlight that the MAPK signaling pathways play a central role during infection. In previous analyses, a central role of MAPKs in the infection of *N. meningitidis* and other pathogens has been described (Sokolova et al., 2004; Dinner et al., 2017; Herold et al., 2021a).

The kinase enrichment analysis pointed to further kinases regulated by *N. meningitidis*, which will need additional experimental confirmation. A kinase whose activity was significantly regulated only after infection with the capsule-deficient mutant is CDK1. This indicates mechanisms that allow the pathogen to interfere with cell proliferation, which have previously been demonstrated in brain endothelial cells (Oosthuysen et al., 2016) as well as epithelial cells (von Papen et al., 2016). Another kinase activity found significantly regulated only by MC58siaD is PRKACA, the catalytic subunit α of the cAMP-dependent protein kinase A (PKA) (Turnham and Scott, 2016). Although bacterial manipulation of host cyclic AMP levels has been described (Serezani et al., 2008), clear evidence for a connection between *N. meningitidis* infection and PKA during infection has not been published yet. Our data indicate a role of cAMP signalling during infection at the BCSFB by *N. meningitidis*, but clarification of the consequences and the mechanisms involved requires further investigation. Kinase activities identified to be regulated by both wild type and mutant *N. meningitidis* in the kinase enrichment analysis are the receptor tyrosine kinase RET and serine-threonine protein kinases Akt1 and Akt2. Functions of RET seem to be mainly involved in development, metabolic diseases and cancer (Takahashi, 2022), therefore it is difficult to conclude a role during infection by *N. meningitidis* at the BCSFB. The Akt signalling network is highly complex and involved in multiple host cell functions (Manning and Toker, 2017), but evidence for a role following challenge with *N. meningitidis* has also not been shown. The described functions of Akt signalling during cellular survival (Song et al., 2005) indicate a role in host cell survival/apoptosis and possibly barrier function following *N. meningitidis* infection of HIBCPP cells.

Taken together, the data presented in this study points towards a stronger impact on the host cell phosphoproteome of HIBCPP cells following infection with the *N. meningitidis* capsule-deficient mutant compared to the wildtype. This corresponds to previous investigations, which revealed higher infection rates of the capsule-deficient strain in HIBCPP cells as well as a differential impact on cellular pathways such as MAPK, dynamin and Src kinases (Borkowski et al., 2014; Herold et al., 2021a; Herold et al., 2021b). Still, it should be considered that at later time points the influence of the wildtype on the host cell proteome could approach that of the capsule-depleted mutant.

## Data availability statement

The mass spectrometry proteomics data have been deposited to the ProteomeXchange Consortium via the PRIDE (Perez-Riverol et al., 2022) partner repository with the dataset identifier PXD038560.

## Author contributions

RH carried out the molecular lab work, participated in the design of the study and drafted the manuscript. LD, WM and CS-G carried out molecular lab work. HI provided material. CS participated in and supervised the design of the study and critically revised the manuscript. HS critically revised the manuscript. All authors contributed to the article and approved the submitted version.

## Supplementary material

Supplementary Table 1 Statistical result of comparison Con vs MC58. A summary of the statistical result of comparison Con vs MC58 showing all data and the results with a p-value ≤ 0.05 and an adjusted p-value ≤ 0.05 after statistical filtering of the data.

Supplementary Table 2 Statistical result of comparison Con vs MC58siaD. A summary of the statistical result of comparison Con vs MC58siaD showing all data and the results with a p-value ≤ 0.05 and an adjusted p-value ≤ 0.05 after statistical filtering of the data.

Supplementary Table 3 Statistical result of comparison MC58 vs MC58siaD. A summary of the statistical result of comparison MC58 vs MC58siaD showing all data and the results with a p-value ≤ 0.05 and an adjusted p-value ≤ 0.05 after statistical filtering of the data.

Supplementary Table 4 Summary of annotated potentially regulated Cellular Components, Molecular Function, Biological Processes, Pathways and active Kinases across groups. Enrichment Analysis were carried out using all regulations with an uncorrected p-value below 0.05. Theses analyses were carried out using the Benjamini-Hochberg Procedure and returning the enriched pathways. The adjusted p-value cut off was set at 0.05.

Supplementary Figure 5
*N. meningitidis* wildtype strain as well as its capsule-deficient mutant activate the MAPK p38 when the basolateral side of the HIBCPP cells is infected for 4h with an MOI 100. Untreated HIBCPP cells (con) were used as negative controls. All biological replicates used in the phosphoproteomic analysis were tested.
