# Corrigendum: The phosphoproteome of choroid plexus epithelial cells following infection with Neisseria meningitidis

**DOI:** 10.3389/fcimb.2023.1249940

**Published:** 2023-08-11

**Authors:** Rosanna Herold, Lea Denzer, Walter Muranyi, Carolin Stump-Guthier, Hiroshi Ishikawa, Horst Schroten, Christian Schwerk

**Affiliations:** ^1^ Pediatric Infectious Diseases, Department of Pediatrics, Medical Faculty Mannheim, Heidelberg University, Mannheim, Germany; ^2^ European Center for Angioscience, Medical Faculty Mannheim, Heidelberg University, Mannheim, Germany; ^3^ Laboratory of Clinical Regenerative Medicine, Department of Neurosurgery, Faculty of Medicine, University of Tsukuba, Tsukuba, Japan

**Keywords:** blood-cerebrospinal fluid barrier, host innate signaling, host pathogen interaction, phosphoproteome, meningitis, *Neisseria meningitidis*

**Text Correction**

In the published article, there was an error. At two places in the text the article published unfortunately did not acknowledge correctly in terms of who performed which work.

A correction has been made to **Materials and methods**, *Infection of HIBCPP cells with N. meningitidis and generation of protein lysates*, paragraph number 1. This sentence previously stated:

“Infection of the HIBCPP cells with the *N. meningitidis* strains MC58 and MC58siaD was described previously (Borkowski et al., 2014). In short, the confluent HIBCPP cells were grown in the inverted culture system and infected with the *N. meningitidis* strains at an MOI of 100 for 4h in DMEM/F12 with 1% FCS and 5 mg/ml insulin. Bacterial infection of the HIBCPP cells was followed by a wash step in PBS and subsequent extraction of whole protein lysate by using modified RIPA buffer (1x RIPA lysis buffer, 50 mM NaF,1 mM Na3VO4, 1 mM PMSF, protease inhibitor cocktail). These lysates were sent to Biogenity (Denmark) for analysis of the phosphoproteome. The infection experiment was performed five times and for each condition, protein lysates of three separate infected filters were pooled.”

The corrected sentence appears below:

“Infection of the HIBCPP cells with the *N. meningitidis* strains MC58 and MC58siaD was described previously (Borkowski et al., 2014). In short, the confluent HIBCPP cells were grown in the inverted culture system and infected with the *N. meningitidis* strains at an MOI of 100 for 4h in DMEM/F12 with 1% FCS and 5 mg/ml insulin. Bacterial infection of the HIBCPP cells was followed by a wash step in PBS and subsequent extraction of whole protein lysate by using modified RIPA buffer (1x RIPA lysis buffer, 50 mM NaF,1 mM Na3VO4, 1 mM PMSF, protease inhibitor cocktail). These lysates were sent to the DTU Proteomics Core facility at the Technical University of Denmark for analysis of the phosphoproteome. The infection experiment was performed five times and for each condition, protein lysates of three separate infected filters were pooled.”

A correction has been made to **Results**, paragraph number 1. This sentence previously stated:

“In order to investigate the influence of the *N. meningitidis* strains on the signal transduction in HIBCPP cells, a phosphoproteomics analysis of the cells after infection with the *N. meningitidis* serogroup B wildtype strain (MC58) and the capsule-deficient mutant (MC58siaD) was carried out in comparison to uninfected HIBCPP cells. For this purpose, the HIBCPP cells were infected in the inverted culture for 4 hours at an MOI of 100 and lysed with the aid of the modified RIPA buffer. We have previously shown that after 4 hours infection from the basolateral side *N. meningitidis* has invaded into HIBCPP cells (Schwerk et al., 2012; Borkowski et al., 2014), and phosphorylation of Erk1/2 and p38 was observed using an MOI of 100 (Herold et al., 2021a). Noteworthy, only a small proportion (around 0.1%) of the meningococcal population is internalized. The samples were processed and analyzed by the company Biogenity (Denmark) for the phosphoproteome analysis. Sample preparation, mass spectrometric analysis and statistical analysis are described in detail in the Material and Methods section.”

The corrected sentence appears below:

“In order to investigate the influence of the *N. meningitidis* strains on the signal transduction in HIBCPP cells, a phosphoproteomics analysis of the cells after infection with the *N. meningitidis* serogroup B wildtype strain (MC58) and the capsule-deficient mutant (MC58siaD) was carried out in comparison to uninfected HIBCPP cells. For this purpose, the HIBCPP cells were infected in the inverted culture for 4 hours at an MOI of 100 and lysed with the aid of the modified RIPA buffer. We have previously shown that after 4 hours infection from the basolateral side *N. meningitidis* has invaded into HIBCPP cells (Schwerk et al., 2012; Borkowski et al., 2014), and phosphorylation of Erk1/2 and p38 was observed using an MOI of 100 (Herold et al., 2021a). Noteworthy, only a small proportion (around 0.1%) of the meningococcal population is internalized. The samples were processed and analyzed by the DTU Proteomics Core facility at the Technical University of Denmark for the phosphoproteome analysis, and data analysis was performed by Biogenity (Denmark). Sample preparation, mass spectrometric analysis and statistical analysis are described in detail in the Material and Methods section.”

The authors apologize for this error and state that this does not change the scientific conclusions of the article in any way. The original article has been updated.

