# Supplementary figures and images for: The phosphoproteome of choroid plexus epithelial cells following infection with Neisseria meningitidis
Source: Front Cell Infect Microbiol. 2023 Mar 31;13:1113528. doi: 10.3389/fcimb.2023.1113528 (PMC10102474; doi:10.3389/fcimb.2023.1113528)

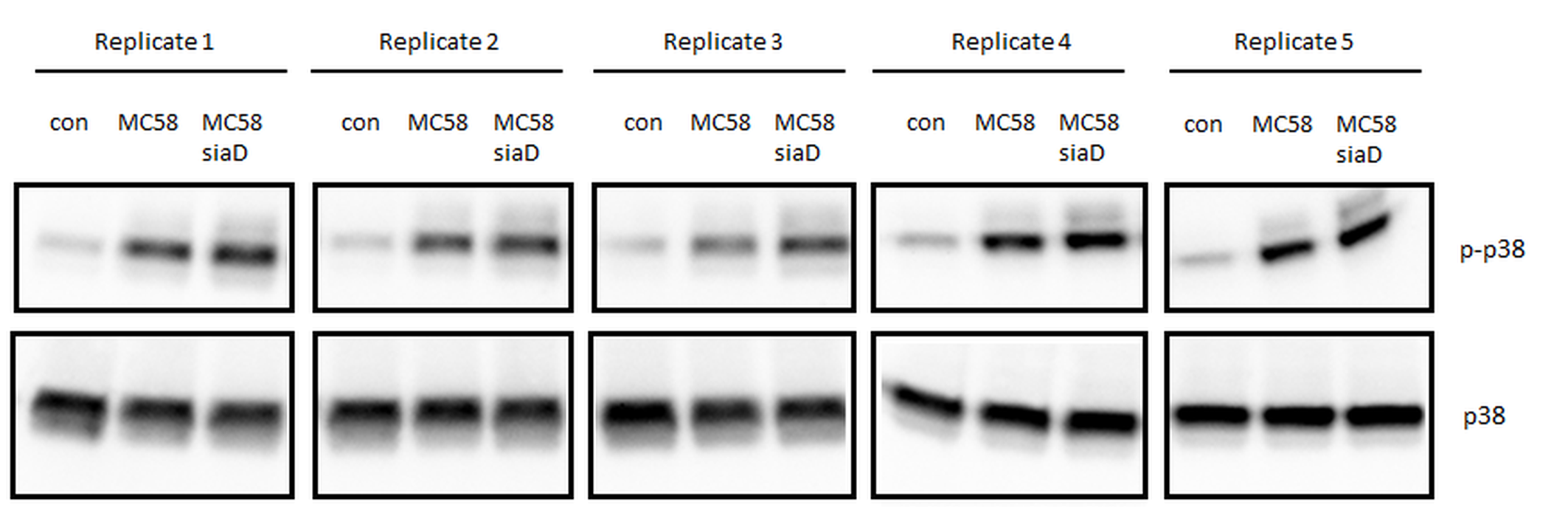

Supplement: Supplementary Figure 5 — N. meningitidis wildtype strain as well as its capsule-deficient mutant activate the MAPK p38 when the basolateral side of the HIBCPP cells is infected for 4h with an MOI 100. Untreated HIBCPP cells (con) were used as negative controls. All biological replicates used in the phosphoproteomic analysis were tested. [file Image_1.tif]
